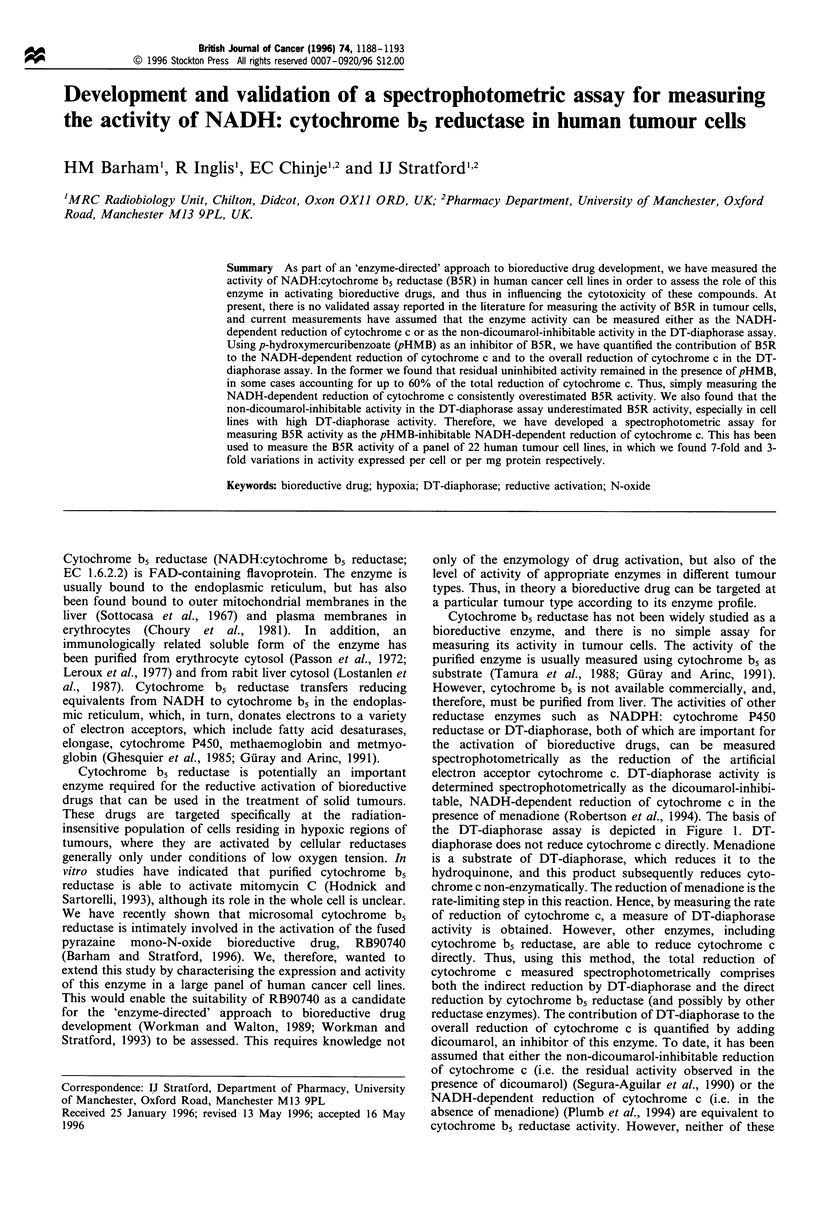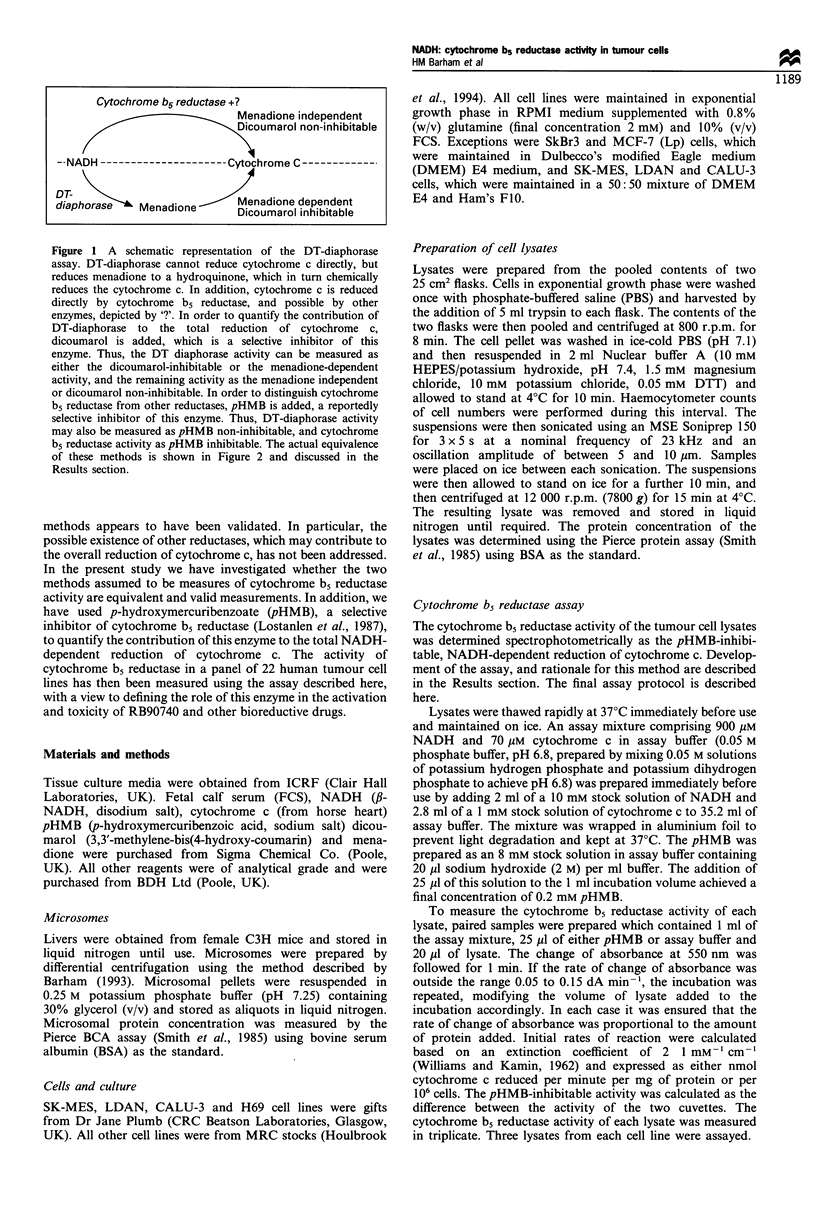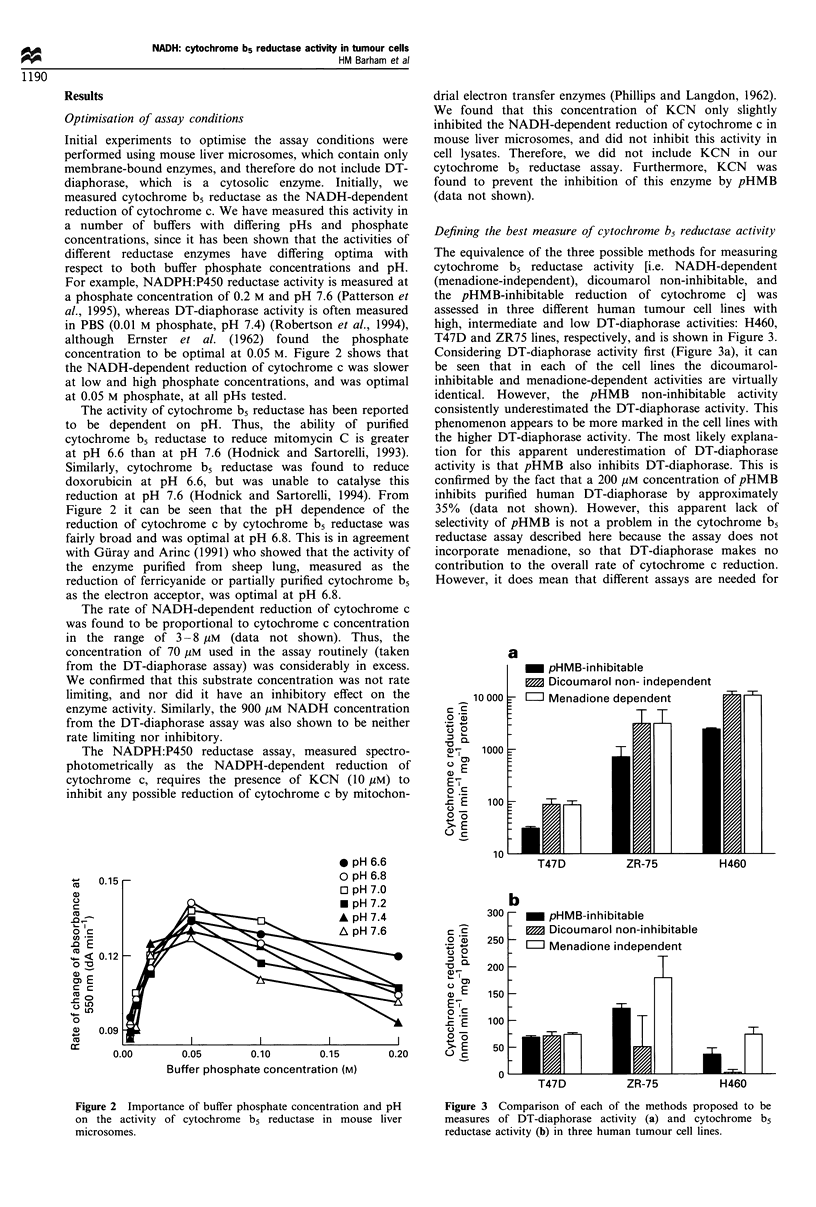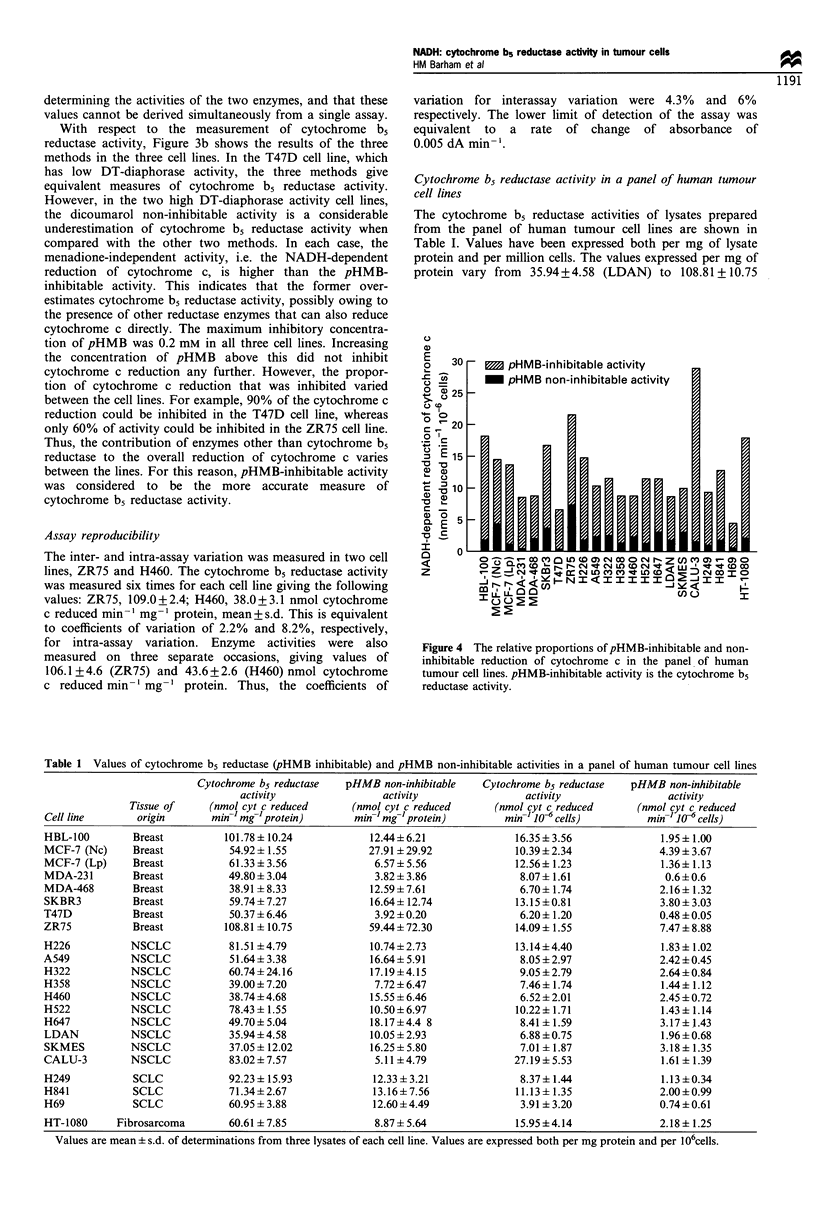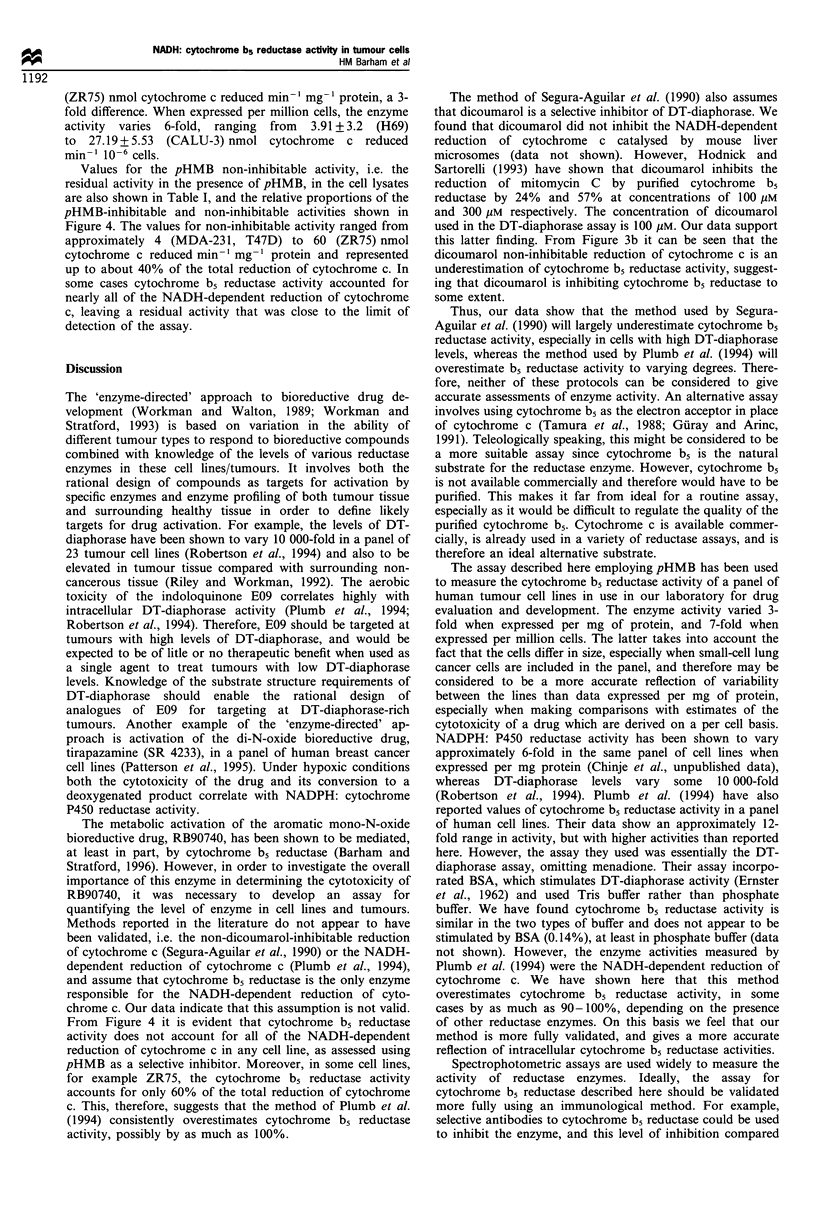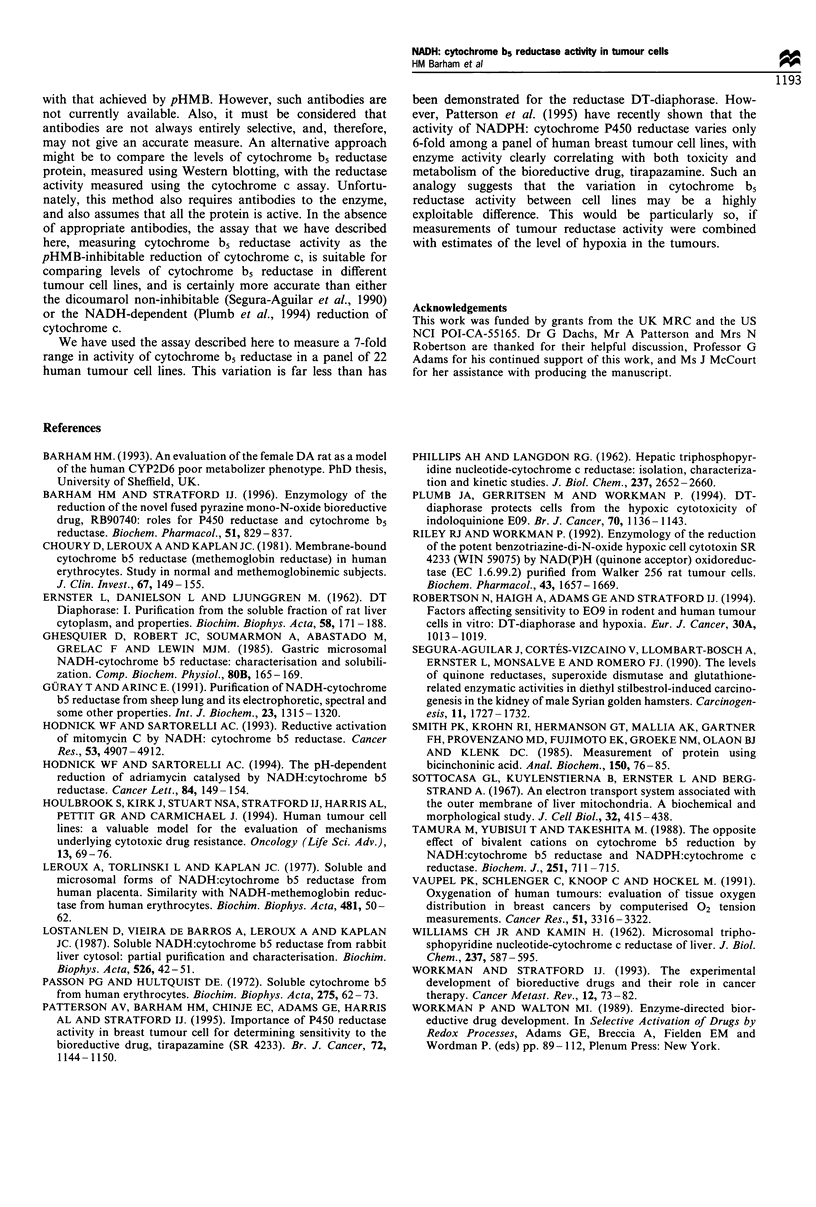# Development and validation of a spectrophotometric assay for measuring the activity of NADH: cytochrome b5 reductase in human tumour cells.

**DOI:** 10.1038/bjc.1996.515

**Published:** 1996-10

**Authors:** H. M. Barham, R. Inglis, E. C. Chinje, I. J. Stratford

**Affiliations:** MRC Radiobiology Unit, Chilton, Didcot, Oxon, UK.

## Abstract

As part of an 'enzyme-directed' approach to bioreductive drug development, we have measured the activity of NADH: cytochrome b5 reductase (B5R) in human cancer cell lines in order to assess the role of this enzyme in activating bioreductive drugs, and thus in influencing the cytotoxicity of these compounds. At present, there is no validated assay reported in the literature for measuring the activity of B5R in tumour cells, and current measurements have assumed that the enzyme activity can be measured either as the NADH-dependent reduction of cytochrome c or as the non-dicoumarol-inhibitable activity in the DT-diaphorase assay. Using p-hydroxymercuribenzoate (pHMB) as an inhibitor of B5R, we have quantified the contribution of B5R to the NADH-dependent reduction of cytochrome c and to the overall reduction of cytochrome c in the DT-diaphorase assay. In the former we found that residual uninhibited activity remained in the presence of pHMB, in some cases accounting for up to 60% of the total reduction of cytochrome c. Thus, simply measuring the NADH-dependent reduction of cytochrome c consistently overestimated B5R activity. We also found that the non-dicoumarol-inhibitable activity in the DT-diaphorase assay underestimated B5R activity, especially in cell lines with high DT-diaphorase activity. Therefore, we have developed a spectrophotometric assay for measuring B5R activity as the pHMB-inhibitable NADH-dependent reduction of cytochrome c. This has been used to measure the B5R activity of a panel of 22 human tumour cell lines, in which we found 7-fold and 3-fold variations in activity expressed per cell or per mg protein respectively.


British Journal of Cancer (1996) 74, 1188-1193
© 1996 Stockton Press All rights reserved 0007-0920/96 $12.00

### Development and validation of a spectrophotometric assay for measuring the activity of NADH: cytochrome $b_5$ reductase in human tumour cells

HM Barham[1], R Inglis[1], EC Chinje[1,2] and IJ Stratford[1,2]

[1]*MRC Radiobiology Unit, Chilton, Didcot, Oxon OX11 ORD, UK;* [2]*Pharmacy Department, University of Manchester, Oxford Road, Manchester M13 9PL, UK.*

**Summary** As part of an 'enzyme-directed' approach to bioreductive drug development, we have measured the activity of NADH:cytochrome $b_5$ reductase (B5R) in human cancer cell lines in order to assess the role of this enzyme in activating bioreductive drugs, and thus in influencing the cytotoxicity of these compounds. At present, there is no validated assay reported in the literature for measuring the activity of B5R in tumour cells, and current measurements have assumed that the enzyme activity can be measured either as the NADH-dependent reduction of cytochrome c or as the non-dicoumarol-inhibitable activity in the DT-diaphorase assay. Using *p*-hydroxymercuribenzoate (*p*HMB) as an inhibitor of B5R, we have quantified the contribution of B5R to the NADH-dependent reduction of cytochrome c and to the overall reduction of cytochrome c in the DT-diaphorase assay. In the former we found that residual uninhibited activity remained in the presence of *p*HMB, in some cases accounting for up to 60% of the total reduction of cytochrome c. Thus, simply measuring the NADH-dependent reduction of cytochrome c consistently overestimated B5R activity. We also found that the non-dicoumarol-inhibitable activity in the DT-diaphorase assay underestimated B5R activity, especially in cell lines with high DT-diaphorase activity. Therefore, we have developed a spectrophotometric assay for measuring B5R activity as the *p*HMB-inhibitable NADH-dependent reduction of cytochrome c. This has been used to measure the B5R activity of a panel of 22 human tumour cell lines, in which we found 7-fold and 3-fold variations in activity expressed per cell or per mg protein respectively.

**Keywords:** bioreductive drug; hypoxia; DT-diaphorase; reductive activation; N-oxide

Cytochrome $b_5$ reductase (NADH:cytochrome $b_5$ reductase; EC 1.6.2.2) is FAD-containing flavoprotein. The enzyme is usually bound to the endoplasmic reticulum, but has also been found bound to outer mitochondrial membranes in the liver (Sottocasa *et al.*, 1967) and plasma membranes in erythrocytes (Choury *et al.*, 1981). In addition, an immunologically related soluble form of the enzyme has been purified from erythrocyte cytosol (Passon *et al.*, 1972; Leroux *et al.*, 1977) and from rabit liver cytosol (Lostanlen *et al.*, 1987). Cytochrome $b_5$ reductase transfers reducing equivalents from NADH to cytochrome $b_5$ in the endoplasmic reticulum, which, in turn, donates electrons to a variety of electron acceptors, which include fatty acid desaturases, elongase, cytochrome P450, methaemoglobin and metmyoglobin (Ghesquier *et al.*, 1985; Güray and Arinc, 1991).

Cytochrome $b_5$ reductase is potentially an important enzyme required for the reductive activation of bioreductive drugs that can be used in the treatment of solid tumours. These drugs are targeted specifically at the radiation-insensitive population of cells residing in hypoxic regions of tumours, where they are activated by cellular reductases generally only under conditions of low oxygen tension. *In vitro* studies have indicated that purified cytochrome $b_5$ reductase is able to activate mitomycin C (Hodnick and Sartorelli, 1993), although its role in the whole cell is unclear. We have recently shown that microsomal cytochrome $b_5$ reductase is intimately involved in the activation of the fused pyrazaine mono-N-oxide bioreductive drug, RB90740 (Barham and Stratford, 1996). We, therefore, wanted to extend this study by characterising the expression and activity of this enzyme in a large panel of human cancer cell lines. This would enable the suitability of RB90740 as a candidate for the 'enzyme-directed' approach to bioreductive drug development (Workman and Walton, 1989; Workman and Stratford, 1993) to be assessed. This requires knowledge not only of the enzymology of drug activation, but also of the level of activity of appropriate enzymes in different tumour types. Thus, in theory a bioreductive drug can be targeted at a particular tumour type according to its enzyme profile.

Cytochrome $b_5$ reductase has not been widely studied as a bioreductive enzyme, and there is no simple assay for measuring its activity in tumour cells. The activity of the purified enzyme is usually measured using cytochrome $b_5$ as substrate (Tamura *et al.*, 1988; Güray and Arinc, 1991). However, cytochrome $b_5$ is not available commercially, and, therefore, must be purified from liver. The activities of other reductase enzymes such as NADPH: cytochrome P450 reductase or DT-diaphorase, both of which are important for the activation of bioreductive drugs, can be measured spectrophotometrically as the reduction of the artificial electron acceptor cytochrome c. DT-diaphorase activity is determined spectrophotometrically as the dicoumarol-inhibitable, NADH-dependent reduction of cytochrome c in the presence of menadione (Robertson *et al.*, 1994). The basis of the DT-diaphorase assay is depicted in Figure 1. DT-diaphorase does not reduce cytochrome c directly. Menadione is a substrate of DT-diaphorase, which reduces it to the hydroquinone, and this product subsequently reduces cytochrome c non-enzymatically. The reduction of menadione is the rate-limiting step in this reaction. Hence, by measuring the rate of reduction of cytochrome c, a measure of DT-diaphorase activity is obtained. However, other enzymes, including cytochrome $b_5$ reductase, are able to reduce cytochrome c directly. Thus, using this method, the total reduction of cytochrome c measured spectrophotometrically comprises both the indirect reduction by DT-diaphorase and the direct reduction by cytochrome $b_5$ reductase (and possibly by other reductase enzymes). The contribution of DT-diaphorase to the overall reduction of cytochrome c is quantified by adding dicoumarol, an inhibitor of this enzyme. To date, it has been assumed that either the non-dicoumarol-inhibitable reduction of cytochrome c (i.e. the residual activity observed in the presence of dicoumarol) (Segura-Aguilar *et al.*, 1990) or the NADH-dependent reduction of cytochrome c (i.e. in the absence of menadione) (Plumb *et al.*, 1994) are equivalent to cytochrome $b_5$ reductase activity. However, neither of these

Correspondence: IJ Stratford, Department of Pharmacy, University of Manchester, Oxford Road, Manchester M13 9PL
Received 25 January 1996; revised 13 May 1996; accepted 16 May 1996

**Figure 1** A schematic representation of the DT-diaphorase assay. DT-diaphorase cannot reduce cytochrome c directly, but reduces menadione to a hydroquinone, which in turn chemically reduces the cytochrome c. In addition, cytochrome c is reduced directly by cytochrome $b_5$ reductase, and possible by other enzymes, depicted by '?'. In order to quantify the contribution of DT-diaphorase to the total reduction of cytochrome c, dicoumarol is added, which is a selective inhibitor of this enzyme. Thus, the DT diaphorase activity can be measured as either the dicoumarol-inhibitable or the menadione-dependent activity, and the remaining activity as the menadione independent or dicoumarol non-inhibitable. In order to distinguish cytochrome $b_5$ reductase from other reductases, *p*HMB is added, a reportedly selective inhibitor of this enzyme. Thus, DT-diaphorase activity may also be measured as *p*HMB non-inhibitable, and cytochrome $b_5$ reductase activity as *p*HMB inhibitable. The actual equivalence of these methods is shown in Figure 2 and discussed in the Results section.

methods appears to have been validated. In particular, the possible existence of other reductases, which may contribute to the overall reduction of cytochrome c, has not been addressed. In the present study we have investigated whether the two methods assumed to be measures of cytochrome $b_5$ reductase activity are equivalent and valid measurements. In addition, we have used *p*-hydroxymercuribenzoate (*p*HMB), a selective inhibitor of cytochrome $b_5$ reductase (Lostanlen *et al.*, 1987), to quantify the contribution of this enzyme to the total NADH-dependent reduction of cytochrome c. The activity of cytochrome $b_5$ reductase in a panel of 22 human tumour cell lines has then been measured using the assay described here, with a view to defining the role of this enzyme in the activation and toxicity of RB90740 and other bioreductive drugs.

### Materials and methods

Tissue culture media were obtained from ICRF (Clair Hall Laboratories, UK). Fetal calf serum (FCS), NADH ($\beta$-NADH, disodium salt), cytochrome c (from horse heart) *p*HMB (*p*-hydroxymercuribenzoic acid, sodium salt) dicoumarol (3,3′-methylene-bis(4-hydroxy-coumarin) and menadione were purchased from Sigma Chemical Co. (Poole, UK). All other reagents were of analytical grade and were purchased from BDH Ltd (Poole, UK).

### *Microsomes*

Livers were obtained from female C3H mice and stored in liquid nitrogen until use. Microsomes were prepared by differential centrifugation using the method described by Barham (1993). Microsomal pellets were resuspended in 0.25 M potassium phosphate buffer (pH 7.25) containing 30% glycerol (v/v) and stored as aliquots in liquid nitrogen. Microsomal protein concentration was measured by the Pierce BCA assay (Smith *et al.*, 1985) using bovine serum albumin (BSA) as the standard.

### *Cells and culture*

SK-MES, LDAN, CALU-3 and H69 cell lines were gifts from Dr Jane Plumb (CRC Beatson Laboratories, Glasgow, UK). All other cell lines were from MRC stocks (Houlbrook *et al.*, 1994). All cell lines were maintained in exponential growth phase in RPMI medium supplemented with 0.8% (w/v) glutamine (final concentration 2 mM) and 10% (v/v) FCS. Exceptions were SkBr3 and MCF-7 (Lp) cells, which were maintained in Dulbecco's modified Eagle medium (DMEM) E4 medium, and SK-MES, LDAN and CALU-3 cells, which were maintained in a 50:50 mixture of DMEM E4 and Ham's F10.

### *Preparation of cell lysates*

Lysates were prepared from the pooled contents of two 25 $cm^2$ flasks. Cells in exponential growth phase were washed once with phosphate-buffered saline (PBS) and harvested by the addition of 5 ml trypsin to each flask. The contents of the two flasks were then pooled and centrifuged at 800 r.p.m. for 8 min. The cell pellet was washed in ice-cold PBS (pH 7.1) and then resuspended in 2 ml Nuclear buffer A (10 mM HEPES/potassium hydroxide, pH 7.4, 1.5 mM magnesium chloride, 10 mM potassium chloride, 0.05 mM DTT) and allowed to stand at 4°C for 10 min. Haemocytometer counts of cell numbers were performed during this interval. The suspensions were then sonicated using an MSE Soniprep 150 for $3\times5$ s at a nominal frequency of 23 kHz and an oscillation amplitude of between 5 and 10 $\mu$m. Samples were placed on ice between each sonication. The suspensions were then allowed to stand on ice for a further 10 min, and then centrifuged at 12 000 r.p.m. (7800 ***g***) for 15 min at 4°C. The resulting lysate was removed and stored in liquid nitrogen until required. The protein concentration of the lysates was determined using the Pierce protein assay (Smith *et al.*, 1985) using BSA as the standard.

### *Cytochrome $b_5$ reductase assay*

The cytochrome $b_5$ reductase activity of the tumour cell lysates was determined spectrophotometrically as the *p*HMB-inhibitable, NADH-dependent reduction of cytochrome c. Development of the assay, and rationale for this method are described in the Results section. The final assay protocol is described here.

Lysates were thawed rapidly at 37°C immediately before use and maintained on ice. An assay mixture comprising 900 $\mu$M NADH and 70 $\mu$M cytochrome c in assay buffer (0.05 M phosphate buffer, pH 6.8, prepared by mixing 0.05 M solutions of potassium hydrogen phosphate and potassium dihydrogen phosphate to achieve pH 6.8) was prepared immediately before use by adding 2 ml of a 10 mM stock solution of NADH and 2.8 ml of a 1 mM stock solution of cytochrome c to 35.2 ml of assay buffer. The mixture was wrapped in aluminium foil to prevent light degradation and kept at 37°C. The *p*HMB was prepared as an 8 mM stock solution in assay buffer containing 20 $\mu$l sodium hydroxide (2 M) per ml buffer. The addition of 25 $\mu$l of this solution to the 1 ml incubation volume achieved a final concentration of 0.2 mM *p*HMB.

To measure the cytochrome $b_5$ reductase activity of each lysate, paired samples were prepared which contained 1 ml of the assay mixture, 25 $\mu$l of either *p*HMB or assay buffer and 20 $\mu$l of lysate. The change of absorbance at 550 nm was followed for 1 min. If the rate of change of absorbance was outside the range 0.05 to 0.15 dA $min^{-1}$, the incubation was repeated, modifying the volume of lysate added to the incubation accordingly. In each case it was ensured that the rate of change of absorbance was proportional to the amount of protein added. Initial rates of reaction were calculated based on an extinction coefficient of 2 1 $mM^{-1}$ $cm^{-1}$ (Williams and Kamin, 1962) and expressed as either nmol cytochrome c reduced per minute per mg of protein or per $10^6$ cells. The *p*HMB-inhibitable activity was calculated as the difference between the activity of the two cuvettes. The cytochrome $b_5$ reductase activity of each lysate was measured in triplicate. Three lysates from each cell line were assayed.

### Results

*Optimisation of assay conditions*

Initial experiments to optimise the assay conditions were performed using mouse liver microsomes, which contain only membrane-bound enzymes, and therefore do not include DT-diaphorase, which is a cytosolic enzyme. Initially, we measured cytochrome $b_5$ reductase as the NADH-dependent reduction of cytochrome c. We have measured this activity in a number of buffers with differing pHs and phosphate concentrations, since it has been shown that the activities of different reductase enzymes have differing optima with respect to both buffer phosphate concentrations and pH. For example, NADPH:P450 reductase activity is measured at a phosphate concentration of 0.2 M and pH 7.6 (Patterson *et al.*, 1995), whereas DT-diaphorase activity is often measured in PBS (0.01 M phosphate, pH 7.4) (Robertson *et al.*, 1994), although Ernster *et al.* (1962) found the phosphate concentration to be optimal at 0.05 M. Figure 2 shows that the NADH-dependent reduction of cytochrome c was slower at low and high phosphate concentrations, and was optimal at 0.05 M phosphate, at all pHs tested.

The activity of cytochrome $b_5$ reductase has been reported to be dependent on pH. Thus, the ability of purified cytochrome $b_5$ reductase to reduce mitomycin C is greater at pH 6.6 than at pH 7.6 (Hodnick and Sartorelli, 1993). Similarly, cytochrome $b_5$ reductase was found to reduce doxorubicin at pH 6.6, but was unable to catalyse this reduction at pH 7.6 (Hodnick and Sartorelli, 1994). From Figure 2 it can be seen that the pH dependence of the reduction of cytochrome c by cytochrome $b_5$ reductase was fairly broad and was optimal at pH 6.8. This is in agreement with Güray and Arinc (1991) who showed that the activity of the enzyme purified from sheep lung, measured as the reduction of ferricyanide or partially purified cytochrome $b_5$ as the electron acceptor, was optimal at pH 6.8.

The rate of NADH-dependent reduction of cytochrome c was found to be proportional to cytochrome c concentration in the range of 3–8 $\mu$M (data not shown). Thus, the concentration of 70 $\mu$M used in the assay routinely (taken from the DT-diaphorase assay) was considerably in excess. We confirmed that this substrate concentration was not rate limiting, and nor did it have an inhibitory effect on the enzyme activity. Similarly, the 900 $\mu$M NADH concentration from the DT-diaphorase assay was also shown to be neither rate limiting nor inhibitory.

The NADPH:P450 reductase assay, measured spectrophotometrically as the NADPH-dependent reduction of cytochrome c, requires the presence of KCN (10 $\mu$M) to inhibit any possible reduction of cytochrome c by mitochondrial electron transfer enzymes (Phillips and Langdon, 1962). We found that this concentration of KCN only slightly inhibited the NADH-dependent reduction of cytochrome c in mouse liver microsomes, and did not inhibit this activity in cell lysates. Therefore, we did not include KCN in our cytochrome $b_5$ reductase assay. Furthermore, KCN was found to prevent the inhibition of this enzyme by *p*HMB (data not shown).

*Defining the best measure of cytochrome $b_5$ reductase activity*

The equivalence of the three possible methods for measuring cytochrome $b_5$ reductase activity [i.e. NADH-dependent (menadione-independent), dicoumarol non-inhibitable, and the *p*HMB-inhibitable reduction of cytochrome c] was assessed in three different human tumour cell lines with high, intermediate and low DT-diaphorase activities: H460, T47D and ZR75 lines, respectively, and is shown in Figure 3. Considering DT-diaphorase activity first (Figure 3a), it can be seen that in each of the cell lines the dicoumarol-inhibitable and menadione-dependent activities are virtually identical. However, the *p*HMB non-inhibitable activity consistently underestimated the DT-diaphorase activity. This phenomenon appears to be more marked in the cell lines with the higher DT-diaphorase activity. The most likely explanation for this apparent underestimation of DT-diaphorase activity is that *p*HMB also inhibits DT-diaphorase. This is confirmed by the fact that a 200 $\mu$M concentration of *p*HMB inhibits purified human DT-diaphorase by approximately 35% (data not shown). However, this apparent lack of selectivity of *p*HMB is not a problem in the cytochrome $b_5$ reductase assay described here because the assay does not incorporate menadione, so that DT-diaphorase makes no contribution to the overall rate of cytochrome c reduction. However, it does mean that different assays are needed for

**Figure 2** Importance of buffer phosphate concentration and pH on the activity of cytochrome $b_5$ reductase in mouse liver microsomes.

**Figure 3** Comparison of each of the methods proposed to be measures of DT-diaphorase activity (**a**) and cytochrome $b_5$ reductase activity (**b**) in three human tumour cell lines.

determining the activities of the two enzymes, and that these values cannot be derived simultaneously from a single assay.

With respect to the measurement of cytochrome $b_5$ reductase activity, Figure 3b shows the results of the three methods in the three cell lines. In the T47D cell line, which has low DT-diaphorase activity, the three methods give equivalent measures of cytochrome $b_5$ reductase activity. However, in the two high DT-diaphorase activity cell lines, the dicoumarol non-inhibitable activity is a considerable underestimation of cytochrome $b_5$ reductase activity when compared with the other two methods. In each case, the menadione-independent activity, i.e. the NADH-dependent reduction of cytochrome c, is higher than the *p*HMB-inhibitable activity. This indicates that the former overestimates cytochrome $b_5$ reductase activity, possibly owing to the presence of other reductase enzymes that can also reduce cytochrome c directly. The maximum inhibitory concentration of *p*HMB was 0.2 mM in all three cell lines. Increasing the concentration of *p*HMB above this did not inhibit cytochrome c reduction any further. However, the proportion of cytochrome c reduction that was inhibited varied between the cell lines. For example, 90% of the cytochrome c reduction could be inhibited in the T47D cell line, whereas only 60% of activity could be inhibited in the ZR75 cell line. Thus, the contribution of enzymes other than cytochrome $b_5$ reductase to the overall reduction of cytochrome c varies between the lines. For this reason, *p*HMB-inhibitable activity was considered to be the more accurate measure of cytochrome $b_5$ reductase activity.

### *Assay reproducibility*

The inter- and intra-assay variation was measured in two cell lines, ZR75 and H460. The cytochrome $b_5$ reductase activity was measured six times for each cell line giving the following values: ZR75, 109.0±2.4; H460, 38.0±3.1 nmol cytochrome c reduced $min^{-1}$ $mg^{-1}$ protein, mean±s.d. This is equivalent to coefficients of variation of 2.2% and 8.2%, respectively, for intra-assay variation. Enzyme activities were also measured on three separate occasions, giving values of 106.1±4.6 (ZR75) and 43.6±2.6 (H460) nmol cytochrome c reduced $min^{-1}$ $mg^{-1}$ protein. Thus, the coefficients of variation for interassay variation were 4.3% and 6% respectively. The lower limit of detection of the assay was equivalent to a rate of change of absorbance of 0.005 dA $min^{-1}$.

### *Cytochrome $b_5$ reductase activity in a panel of human tumour cell lines*

The cytochrome $b_5$ reductase activities of lysates prepared from the panel of human tumour cell lines are shown in Table I. Values have been expressed both per mg of lysate protein and per million cells. The values expressed per mg of protein vary from 35.94±4.58 (LDAN) to 108.81±10.75

**Figure 4** The relative proportions of *p*HMB-inhibitable and non-inhibitable reduction of cytochrome c in the panel of human tumour cell lines. *p*HMB-inhibitable activity is the cytochrome $b_5$ reductase activity.

**Table 1** Values of cytochrome $b_5$ reductase (*p*HMB inhibitable) and *p*HMB non-inhibitable activities in a panel of human tumour cell lines

| *Cell line* | *Tissue of origin* | *Cytochrome $b_5$ reductase activity (nmol cyt c reduced $min^{-1}$ $mg^{-1}$ protein)* | *pHMB non-inhibitable activity (nmol cyt c reduced $min^{-1}$ $mg^{-1}$ protein)* | *Cytochrome $b_5$ reductase activity (nmol cyt c reduced $min^{-1}$ $10^{-6}$ cells)* | *pHMB non-inhibitable activity (nmol cyt c reduced $min^{-1}$ $10^{-6}$ cells)* |
|---|---|---|---|---|---|
| HBL-100 | Breast | 101.78±10.24 | 12.44±6.21 | 16.35±3.56 | 1.95±1.00 |
| MCF-7 (Nc) | Breast | 54.92±1.55 | 27.91±29.92 | 10.39±2.34 | 4.39±3.67 |
| MCF-7 (Lp) | Breast | 61.33±3.56 | 6.57±5.56 | 12.56±1.23 | 1.36±1.13 |
| MDA-231 | Breast | 49.80±3.04 | 3.82±3.86 | 8.07±1.61 | 0.6±0.6 |
| MDA-468 | Breast | 38.91±8.33 | 12.59±7.61 | 6.70±1.74 | 2.16±1.32 |
| SKBR3 | Breast | 59.74±7.27 | 16.64±12.74 | 13.15±0.81 | 3.80±3.03 |
| T47D | Breast | 50.37±6.46 | 3.92±0.20 | 6.20±1.20 | 0.48±0.05 |
| ZR75 | Breast | 108.81±10.75 | 59.44±72.30 | 14.09±1.55 | 7.47±8.88 |
| H226 | NSCLC | 81.51±4.79 | 10.74±2.73 | 13.14±4.40 | 1.83±1.02 |
| A549 | NSCLC | 51.64±3.38 | 16.64±5.91 | 8.05±2.97 | 2.42±0.45 |
| H322 | NSCLC | 60.74±24.16 | 17.19±4.15 | 9.05±2.79 | 2.64±0.84 |
| H358 | NSCLC | 39.00±7.20 | 7.72±6.47 | 7.46±1.74 | 1.44±1.12 |
| H460 | NSCLC | 38.74±4.68 | 15.55±6.46 | 6.52±2.01 | 2.45±0.72 |
| H522 | NSCLC | 78.43±1.55 | 10.50±6.97 | 10.22±1.71 | 1.43±1.14 |
| H647 | NSCLC | 49.70±5.04 | 18.17±4.4 8 | 8.41±1.59 | 3.17±1.43 |
| LDAN | NSCLC | 35.94±4.58 | 10.05±2.93 | 6.88±0.75 | 1.96±0.68 |
| SKMES | NSCLC | 37.05±12.02 | 16.25±5.80 | 7.01±1.87 | 3.18±1.35 |
| CALU-3 | NSCLC | 83.02±7.57 | 5.11±4.79 | 27.19±5.53 | 1.61±1.39 |
| H249 | SCLC | 92.23±15.93 | 12.33±3.21 | 8.37±1.44 | 1.13±0.34 |
| H841 | SCLC | 71.34±2.67 | 13.16±7.56 | 11.13±1.35 | 2.00±0.99 |
| H69 | SCLC | 60.95±3.88 | 12.60±4.49 | 3.91±3.20 | 0.74±0.61 |
| HT-1080 | Fibrosarcoma | 60.61±7.85 | 8.87±5.64 | 15.95±4.14 | 2.18±1.25 |

Values are mean±s.d. of determinations from three lysates of each cell line. Values are expressed both per mg protein and per $10^6$cells.

(ZR75) nmol cytochrome c reduced $min^{-1}$ $mg^{-1}$ protein, a 3-fold difference. When expressed per million cells, the enzyme activity varies 6-fold, ranging from $3.91 \pm 3.2$ (H69) to $27.19 \pm 5.53$ (CALU-3) nmol cytochrome c reduced $min^{-1}$ $10^{-6}$ cells.

Values for the *p*HMB non-inhibitable activity, i.e. the residual activity in the presence of *p*HMB, in the cell lysates are also shown in Table I, and the relative proportions of the *p*HMB-inhibitable and non-inhibitable activities shown in Figure 4. The values for non-inhibitable activity ranged from approximately 4 (MDA-231, T47D) to 60 (ZR75) nmol cytochrome c reduced $min^{-1}$ $mg^{-1}$ protein and represented up to about 40% of the total reduction of cytochrome c. In some cases cytochrome $b_5$ reductase activity accounted for nearly all of the NADH-dependent reduction of cytochrome c, leaving a residual activity that was close to the limit of detection of the assay.

### Discussion

The 'enzyme-directed' approach to bioreductive drug development (Workman and Walton, 1989; Workman and Stratford, 1993) is based on variation in the ability of different tumour types to respond to bioreductive compounds combined with knowledge of the levels of various reductase enzymes in these cell lines/tumours. It involves both the rational design of compounds as targets for activation by specific enzymes and enzyme profiling of both tumour tissue and surrounding healthy tissue in order to define likely targets for drug activation. For example, the levels of DT-diaphorase have been shown to vary 10 000-fold in a panel of 23 tumour cell lines (Robertson *et al.*, 1994) and also to be elevated in tumour tissue compared with surrounding non-cancerous tissue (Riley and Workman, 1992). The aerobic toxicity of the indoloquinone E09 correlates highly with intracellular DT-diaphorase activity (Plumb *et al.*, 1994; Robertson *et al.*, 1994). Therefore, E09 should be targeted at tumours with high levels of DT-diaphorase, and would be expected to be of litle or no therapeutic benefit when used as a single agent to treat tumours with low DT-diaphorase levels. Knowledge of the substrate structure requirements of DT-diaphorase should enable the rational design of analogues of E09 for targeting at DT-diaphorase-rich tumours. Another example of the 'enzyme-directed' approach is activation of the di-N-oxide bioreductive drug, tirapazamine (SR 4233), in a panel of human breast cancer cell lines (Patterson *et al.*, 1995). Under hypoxic conditions both the cytotoxicity of the drug and its conversion to a deoxygenated product correlate with NADPH: cytochrome P450 reductase activity.

The metabolic activation of the aromatic mono-N-oxide bioreductive drug, RB90740, has been shown to be mediated, at least in part, by cytochrome $b_5$ reductase (Barham and Stratford, 1996). However, in order to investigate the overall importance of this enzyme in determining the cytotoxicity of RB90740, it was necessary to develop an assay for quantifying the level of enzyme in cell lines and tumours. Methods reported in the literature do not appear to have been validated, i.e. the non-dicoumarol-inhibitable reduction of cytochrome c (Segura-Aguilar *et al.*, 1990) or the NADH-dependent reduction of cytochrome c (Plumb *et al.*, 1994), and assume that cytochrome $b_5$ reductase is the only enzyme responsible for the NADH-dependent reduction of cytochrome c. Our data indicate that this assumption is not valid. From Figure 4 it is evident that cytochrome $b_5$ reductase activity does not account for all of the NADH-dependent reduction of cytochrome c in any cell line, as assessed using *p*HMB as a selective inhibitor. Moreover, in some cell lines, for example ZR75, the cytochrome $b_5$ reductase activity accounts for only 60% of the total reduction of cytochrome c. This, therefore, suggests that the method of Plumb *et al.* (1994) consistently overestimates cytochrome $b_5$ reductase activity, possibly by as much as 100%.

The method of Segura-Aguilar *et al.* (1990) also assumes that dicoumarol is a selective inhibitor of DT-diaphorase. We found that dicoumarol did not inhibit the NADH-dependent reduction of cytochrome c catalysed by mouse liver microsomes (data not shown). However, Hodnick and Sartorelli (1993) have shown that dicoumarol inhibits the reduction of mitomycin C by purified cytochrome $b_5$ reductase by 24% and 57% at concentrations of 100 $\mu$M and 300 $\mu$M respectively. The concentration of dicoumarol used in the DT-diaphorase assay is 100 $\mu$M. Our data support this latter finding. From Figure 3b it can be seen that the dicoumarol non-inhibitable reduction of cytochrome c is an underestimation of cytochrome $b_5$ reductase activity, suggesting that dicoumarol is inhibiting cytochrome $b_5$ reductase to some extent.

Thus, our data show that the method used by Segura-Aguilar *et al.* (1990) will largely underestimate cytochrome $b_5$ reductase activity, especially in cells with high DT-diaphorase levels, whereas the method used by Plumb *et al.* (1994) will overestimate $b_5$ reductase activity to varying degrees. Therefore, neither of these protocols can be considered to give accurate assessments of enzyme activity. An alternative assay involves using cytochrome $b_5$ as the electron acceptor in place of cytochrome c (Tamura *et al.*, 1988; Güray and Arinc, 1991). Teleologically speaking, this might be considered to be a more suitable assay since cytochrome $b_5$ is the natural substrate for the reductase enzyme. However, cytochrome $b_5$ is not available commercially and therefore would have to be purified. This makes it far from ideal for a routine assay, especially as it would be difficult to regulate the quality of the purified cytochrome $b_5$. Cytochrome c is available commercially, is already used in a variety of reductase assays, and is therefore an ideal alternative substrate.

The assay described here employing *p*HMB has been used to measure the cytochrome $b_5$ reductase activity of a panel of human tumour cell lines in use in our laboratory for drug evaluation and development. The enzyme activity varied 3-fold when expressed per mg of protein, and 7-fold when expressed per million cells. The latter takes into account the fact that the cells differ in size, especially when small-cell lung cancer cells are included in the panel, and therefore may be considered to be a more accurate reflection of variability between the lines than data expressed per mg of protein, especially when making comparisons with estimates of the cytotoxicity of a drug which are derived on a per cell basis. NADPH: P450 reductase activity has been shown to vary approximately 6-fold in the same panel of cell lines when expressed per mg protein (Chinje *et al.*, unpublished data), whereas DT-diaphorase levels vary some 10 000-fold (Robertson *et al.*, 1994). Plumb *et al.* (1994) have also reported values of cytochrome $b_5$ reductase activity in a panel of human cell lines. Their data show an approximately 12-fold range in activity, but with higher activities than reported here. However, the assay they used was essentially the DT-diaphorase assay, omitting menadione. Their assay incorporated BSA, which stimulates DT-diaphorase activity (Ernster *et al.*, 1962) and used Tris buffer rather than phosphate buffer. We have found cytochrome $b_5$ reductase activity is similar in the two types of buffer and does not appear to be stimulated by BSA (0.14%), at least in phosphate buffer (data not shown). However, the enzyme activities measured by Plumb *et al.* (1994) were the NADH-dependent reduction of cytochrome c. We have shown here that this method overestimates cytochrome $b_5$ reductase activity, in some cases by as much as 90–100%, depending on the presence of other reductase enzymes. On this basis we feel that our method is more fully validated, and gives a more accurate reflection of intracellular cytochrome $b_5$ reductase activities.

Spectrophotometric assays are used widely to measure the activity of reductase enzymes. Ideally, the assay for cytochrome $b_5$ reductase described here should be validated more fully using an immunological method. For example, selective antibodies to cytochrome $b_5$ reductase could be used to inhibit the enzyme, and this level of inhibition compared with that achieved by *p*HMB. However, such antibodies are not currently available. Also, it must be considered that antibodies are not always entirely selective, and, therefore, may not give an accurate measure. An alternative approach might be to compare the levels of cytochrome $b_5$ reductase protein, measured using Western blotting, with the reductase activity measured using the cytochrome c assay. Unfortunately, this method also requires antibodies to the enzyme, and also assumes that all the protein is active. In the absence of appropriate antibodies, the assay that we have described here, measuring cytochrome $b_5$ reductase activity as the *p*HMB-inhibitable reduction of cytochrome c, is suitable for comparing levels of cytochrome $b_5$ reductase in different tumour cell lines, and is certainly more accurate than either the dicoumarol non-inhibitable (Segura-Aguilar *et al.*, 1990) or the NADH-dependent (Plumb *et al.*, 1994) reduction of cytochrome c.

We have used the assay described here to measure a 7-fold range in activity of cytochrome $b_5$ reductase in a panel of 22 human tumour cell lines. This variation is far less than has been demonstrated for the reductase DT-diaphorase. However, Patterson *et al.* (1995) have recently shown that the activity of NADPH: cytochrome P450 reductase varies only 6-fold among a panel of human breast tumour cell lines, with enzyme activity clearly correlating with both toxicity and metabolism of the bioreductive drug, tirapazamine. Such an analogy suggests that the variation in cytochrome $b_5$ reductase activity between cell lines may be a highly exploitable difference. This would be particularly so, if measurements of tumour reductase activity were combined with estimates of the level of hypoxia in the tumours.

**Acknowledgements**
This work was funded by grants from the UK MRC and the US NCI POI-CA-55165. Dr G Dachs, Mr A Patterson and Mrs N Robertson are thanked for their helpful discussion, Professor G Adams for his continued support of this work, and Ms J McCourt for her assistance with producing the manuscript.

### References

BARHAM HM. (1993). An evaluation of the female DA rat as a model of the human CYP2D6 poor metabolizer phenotype. PhD thesis, University of Sheffield, UK.

BARHAM HM AND STRATFORD IJ. (1996). Enzymology of the reduction of the novel fused pyrazine mono-N-oxide bioreductive drug, RB90740: roles for P450 reductase and cytochrome $b_5$ reductase. *Biochem. Pharmacol.*, **51**, 829–837.

CHOURY D, LEROUX A AND KAPLAN JC. (1981). Membrane-bound cytochrome b5 reductase (methemoglobin reductase) in human erythrocytes. Study in normal and methemoglobinemic subjects. *J. Clin. Invest.*, **67**, 149–155.

ERNSTER L, DANIELSON L AND LJUNGGREN M. (1962). DT Diaphorase: I. Purification from the soluble fraction of rat liver cytoplasm, and properties. *Biochim. Biophys. Acta*, **58**, 171–188.

GHESQUIER D, ROBERT JC, SOUMARMON A, ABASTADO M, GRELAC F AND LEWIN MJM. (1985). Gastric microsomal NADH-cytochrome b5 reductase: characterisation and solubilization. *Comp. Biochem. Physiol.*, **80B**, 165–169.

GÜRAY T AND ARINC E. (1991). Purification of NADH-cytochrome b5 reductase from sheep lung and its electrophoretic, spectral and some other properties. *Int. J. Biochem.*, **23**, 1315–1320.

HODNICK WF AND SARTORELLI AC. (1993). Reductive activation of mitomycin C by NADH: cytochrome b5 reductase. *Cancer Res.*, **53**, 4907–4912.

HODNICK WF AND SARTORELLI AC. (1994). The pH-dependent reduction of adriamycin catalysed by NADH:cytochrome b5 reductase. *Cancer Lett.*, **84**, 149–154.

HOULBROOK S, KIRK J, STUART NSA, STRATFORD IJ, HARRIS AL, PETTIT GR AND CARMICHAEL J. (1994). Human tumour cell lines: a valuable model for the evaluation of mechanisms underlying cytotoxic drug resistance. *Oncology (Life Sci. Adv.)*, **13**, 69–76.

LEROUX A, TORLINSKI L AND KAPLAN JC. (1977). Soluble and microsomal forms of NADH:cytochrome b5 reductase from human placenta. Similarity with NADH-methemoglobin reductase from human erythrocytes. *Biochim. Biophys. Acta*, **481**, 50–62.

LOSTANLEN D, VIEIRA DE BARROS A, LEROUX A AND KAPLAN JC. (1987). Soluble NADH:cytochrome b5 reductase from rabbit liver cytosol: partial purification and characterisation. *Biochim. Biophys. Acta*, **526**, 42–51.

PASSON PG AND HULTQUIST DE. (1972). Soluble cytochrome b5 from human erythrocytes. *Biochim. Biophys. Acta*, **275**, 62–73.

PATTERSON AV, BARHAM HM, CHINJE EC, ADAMS GE, HARRIS AL AND STRATFORD IJ. (1995). Importance of P450 reductase activity in breast tumour cell for determining sensitivity to the bioreductive drug, tirapazamine (SR 4233). *Br. J. Cancer*, **72**, 1144–1150.

PHILLIPS AH AND LANGDON RG. (1962). Hepatic triphosphopyridine nucleotide-cytochrome c reductase: isolation, characterization and kinetic studies. *J. Biol. Chem.*, **237**, 2652–2660.

PLUMB JA, GERRITSEN M AND WORKMAN P. (1994). DT-diaphorase protects cells from the hypoxic cytotoxicity of indoloquinione E09. *Br. J. Cancer*, **70**, 1136–1143.

RILEY RJ AND WORKMAN P. (1992). Enzymology of the reduction of the potent benzotriazine-di-N-oxide hypoxic cell cytotoxin SR 4233 (WIN 59075) by NAD(P)H (quinone acceptor) oxidoreductase (EC 1.6.99.2) purified from Walker 256 rat tumour cells. *Biochem. Pharmacol.*, **43**, 1657–1669.

ROBERTSON N, HAIGH A, ADAMS GE AND STRATFORD IJ. (1994). Factors affecting sensitivity to EO9 in rodent and human tumour cells in vitro: DT-diaphorase and hypoxia. *Eur. J. Cancer*, **30A**, 1013–1019.

SEGURA-AGUILAR J, CORTÉS-VIZCAINO V, LLOMBART-BOSCH A, ERNSTER L, MONSALVE E AND ROMERO FJ. (1990). The levels of quinone reductases, superoxide dismutase and glutathione-related enzymatic activities in diethyl stilbestrol-induced carcinogenesis in the kidney of male Syrian golden hamsters. *Carcinogenesis*, **11**, 1727–1732.

SMITH PK, KROHN RI, HERMANSON GT, MALLIA AK, GARTNER FH, PROVENZANO MD, FUJIMOTO EK, GROEKE NM, OLAON BJ AND KLENK DC. (1985). Measurement of protein using bicinchoninic acid. *Anal. Biochem.*, **150**, 76–85.

SOTTOCASA GL, KUYLENSTIERNA B, ERNSTER L AND BERGSTRAND A. (1967). An electron transport system associated with the outer membrane of liver mitochondria. A biochemical and morphological study. *J. Cell Biol.*, **32**, 415–438.

TAMURA M, YUBISUI T AND TAKESHITA M. (1988). The opposite effect of bivalent cations on cytochrome b5 reduction by NADH:cytochrome b5 reductase and NADPH:cytochrome c reductase. *Biochem. J.*, **251**, 711–715.

VAUPEL PK, SCHLENGER C, KNOOP C AND HOCKEL M. (1991). Oxygenation of human tumours: evaluation of tissue oxygen distribution in breast cancers by computerised $O_2$ tension measurements. *Cancer Res.*, **51**, 3316–3322.

WILLIAMS CH JR AND KAMIN H. (1962). Microsomal triphosphopyridine nucleotide-cytochrome c reductase of liver. *J. Biol. Chem.*, **237**, 587–595.

WORKMAN AND STRATFORD IJ. (1993). The experimental development of bioreductive drugs and their role in cancer therapy. *Cancer Metast. Rev.*, **12**, 73–82.

WORKMAN P AND WALTON MI. (1989). Enzyme-directed bioreductive drug development. In *Selective Activation of Drugs by Redox Processes*, Adams GE, Breccia A, Fielden EM and Wordman P. (eds) pp. 89–112, Plenum Press: New York.